# Dynamic changes of facial skeletal fractures with time

**DOI:** 10.1038/s41598-020-60725-9

**Published:** 2020-03-04

**Authors:** Bao-Hai Yu, Shu-Man Han, Tao Sun, Zhe Guo, Lei Cao, Hui-Zhao Wu, Yun-Heng Shi, Jin-Xu Wen, Wen-Juan Wu, Bu-Lang Gao

**Affiliations:** Department of Radiology, the Third Hospital, Hebei Medical University, Shijiazhuang, China

**Keywords:** Trauma, Bone imaging

## Abstract

To investigate the characteristics of imaging changes with time of facial fractures, patients with facial fractures who had computed tomographic scan were enrolled including 500 patients who were divided into six groups based on the time of scanning: super early (<3 d), early (4–7 d), early-to-medium (8–14 d), medium (15–21d), medium-to-late (22d–2 months) and late stage (>2 months). The data were compared and analyzed. Forty two patients with frontal bone fractures had high-energy impact as the reason of fractures. The fracture line was clear and sharp within one week but blunt and sclerotic due to bone absorption at 2–3 weeks, and might exist for a long time. All patients had soft tissue swelling and paranasal sinus effusion at 1–2 weeks after injury. Air might gather in the adjacent soft tissues and/or intracranially within 3 days of injury if the fracture involved the frontal or other sinuses. Twelve of the 42 patients (28.6%) had intracranial hematoma, and five (11.9%) had epidural effusion. Subarachnoid hemorrhage was mostly absorbed within one week while epidural hematoma was completely absorbed over 3 weeks. Significant changes (P < 0.05) in the fracture lines, effusion of paranasal sinuses, soft tissue swelling and pneumocephalus were observed during the study period. For patients with medial orbital wall fractures, the fracture line was sharp and clear at early stages with concurrent sphenoid sinus effusion, and the fracture line became depressed 3 weeks later with disappearance of sphenoid sinus effusion. Significant changes (P < 0.05) were observed in the sharp fracture line, soft tissue swelling, sphenoid sinus effusion and smooth depression at fracture sites. For nasal fractures, the fracture line was sharp and clear at early stages with concurrent soft tissue swelling which disappeared one week later. The fracture line became smooth three weeks later. A significant (P < 0.05) difference was demonstrated in the changes of fracture line and soft tissue swelling with time. In conclusion, facial fractures have some dynamic alterations with time and identification of these characteristics may help reaching a correct clinical diagnosis with regard to fracture severity and time.

## Introduction

Facial bone fractures are very common and probably related to other life-threatening situations, like traumatic cerebral injuries, and this type of fracture may be complex resulting in serious intracranial complications^1^. Facial fractures are usually caused by high-energy impact, especially traffic accidents, accounting for 24% of all traumatic patients with facial injuries and requiring prompt diagnosis of fractures and soft tissue injuries so that emergency interventions can be performed immediately^2,3^. An increasing number of patients with facial fractures are hospitalized each year, and proper diagnosis and management is the key to prevention of lasting consequences such as permanent asymmetry, malocclusion, disfiguration and enophthalmos^4–6^. High-energy impact injuries are the most common etiology of facial fractures ranging 49–53% for orbital roof fractures reported in the literature^7^. Hospitalizations due to facial skeletal fractures can incur a significant burden in both finances and lost days from either work or school. Clinical symptoms and medical imaging examination should be combined to determine the severity and time of injury for proper management. Some patients may have old injuries or fractures which should be properly identified. Imaging evaluation of traumatic injury of the facial skeleton is challenging because of the complexity of the facial skeleton anatomy together with numerous bony structures presented on plain radiographs which may be helpful in assessing isolated facial injuries^8,9^. Currently, computed tomography (CT) has been widely applied in the diagnosis of facial skeletal fractures as the gold standard imaging method and has revolutionized quick and precise evaluation of neck and craniofacial fractures in those patients with serious head and neck trauma^1,3,10^. Compared with clinical examination and plain X-ray radiography with linear tomography, CT scan is the most accurate tool for diagnosis of facial fractures^3,11^. High definition CT scanners can find even small fractures of the facial skeleton. Scanning with multidetector CT of the facial skeleton has emerged as the principal diagnostic method due to its ease of positioning of the patient, minimization of patient cooperation and creation of multiplanar images of reconstruction^8,9^, allowing for precise and efficient diagnosis of head and neck injury, especially in the maxillofacial region^8,9,12,13^. Moreover, CT imaging can facilitate planning for surgical treatment of facial skeleton fracture and permit precise evaluation of bone stability, rotation and displacement of fragments of bone^8^. The signs of facial fractures can change with time on CT scan, and this study was performed to identify the changes of facial fractures with time on CT imaging so as to provide evidence for judging old or new injuries and to guide imaging follow-up.

## Materials and Methods

This study was approved by the ethics committee of the Third Hospital of Hebei Medical University with all patients or their legal guardians given the signed informed consent for study participation. All methods were performed in accordance with the relevant guidelines and regulations. Inclusion criteria were consecutive patients with facial fractures which had not been treated with surgery, with a clear history of disease, with multiple imaging examinations but no old injuries. Exclusion criteria were patients with facial fractures which had been treated with surgery, with only one imaging examination, unclear history of disease and old injuries. Between January 2016 and March 2017, 500 patients with facial skeletal fractures were enrolled in this study, including 369 male and 131 female patients with a male to female ratio of 2.82:1 and an age range of 5–72 years (mean 40.24 ± 18.71 years). Among all the patients, 264 (52.8%) patients had traffic accidents, 92 (18.4%) had accidental fall, 31 (6.2%) had violent assaults, and 113 (22.6%) had other accidental injuries. Based on the time interval of injury and CT scanning, these patients were divided into six groups: super early stage (<3 d), early stage (4–7 d), early-to-medium stage (8–14 d), medium stage (15–21 d), medium-to-late stage (22 d-2 months) and late stage (>2 months).

The Siemens Somatom Sensation 64 or 128 slice scanners were used for examination with the patients in the supine position, and the canthomeatal line or auditory orbital line was used as the base line for scanning. The scanning parameters were tube voltage 120 kV, automatic adjustment of tube current (range 74–94 mA) and matrix 512 × 512. The scanning and reconstruction interval was ≤1 mm for scanning of the nasal area and ≤2 mm for cranial base and orbital wall. Two senior radiologists who were blinded to the clinical data judged the CT images, and a third one was involved when in disagreement. The observed parameters were clarity of the fracture line, bluntness or hardening at the edge of fracture, swelling of pneumatosis in adjacent soft tissues, effusion in adjacent sinuses and injury and hemorrhage of adjacent brain tissues including pneumatosis, epidural or subdural hematoma, subdural collection of fluid, subarachnoid hemorrhage and brain contusion and laceration. In addition, the age, sex, mechanism of injury, single or multiple fractures, number of imaging examinations and outcomes of the patients were also evaluated.

### Statistical analysis

The statistical analysis software SPSS 19.0 (IBM, Chicago, IL, USA) was used for analysis. Enumeration data were expressed as rate (%), and the Chi-square test was used to compare imaging signs between different stages and between the first and latter three stages. The P value of <0.05 was set as statistically significant.

## Results

### Type of fractures

Simple fractures were in 276 cases (55.2%) including nasal and/or maxillofacial fractures in 165 cases (33.0%), nasal and/or orbital fracture in 67 cases (13.4%), single mandible fracture in 34 (6.8%) and single fracture of zygomatic arch in 10 (2.0%). There were 121 (24.4%) cases with fractures in the zygomatic-maxillary complex and 103 (20.6%) cases with multiple facial fractures. Among all the facial fractures, the fracture involved the nasal and/or maxillofacial bone in 229 cases (45.8%), orbital bone in 134 cases (26.8%) and frontal bone in 42 cases (8.4%).

### Frontal fractures

Forty-two patients with frontal bone fractures also had other facial or cranial fractures including 29 cases with concomitant fractures in other body parts. The fracture in this group was all caused by high-energy impact injury including traffic accidents and falls. A total of 148 CT scanning was performed in these 42 patients (mean 3.52 per person). A linear fracture of the frontal bone existed in 18 cases (34%) involving the frontal sinus in 21 cases (50%) and cranial base in 17 (40.5%).

**Figure 1:**
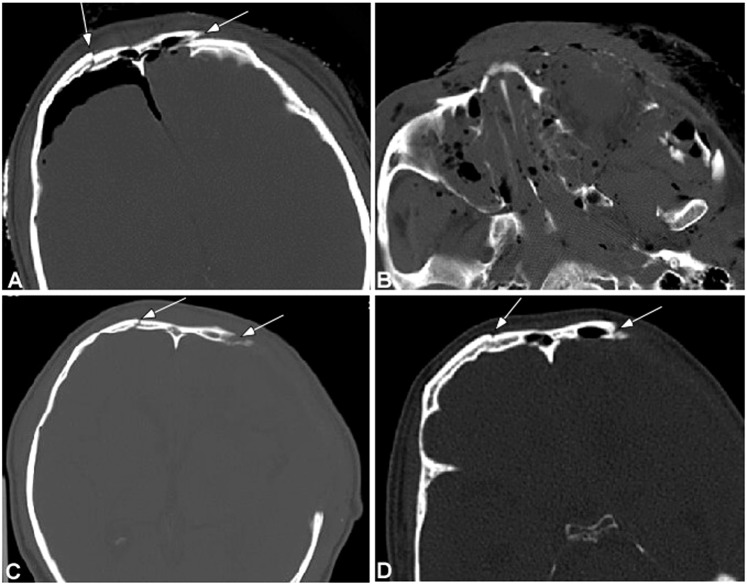
A male patient of 45 years old had a traffic accident and was diagnosed as having multiple facial fractures, left epidural hematoma, contusion and laceration in the left frontal lobe and left optic nerve injury. (**A**) On the day of injury, the frontal fracture lines were sharp and clear (arrows) involving bilateral frontal sinus walls with intracranial air collection. (**B**) Comminuted fractures were shown in the maxillofacial bones with maxillary sinus effusion, air collection in the adjacent soft tissues and swelling of soft tissues. (**C**) Follow-up scanning two weeks later demonstrated blunt edge of the frontal bone fracture lines (arrows) indicating bone absorption. (**D**) Three months later, the fracture lines were further blunted (arrow). Bone defect was shown in (**C**,**D**) after procedure.

The fracture line was clear and sharp within one week of injury, blunt and sclerosed at the fracture edge 2–3 weeks later, and could be observed over 2 months. Within one to two weeks of injury, obvious swelling existed in adjacent soft tissues, with effusion and mucosal thickening in paranasal sinuses. The effusion in the paranasal sinuses could last a long time. Within three days of injury, air could be observed in the adjacent soft tissues and/or intracranially when the fracture involved the frontal sinus, and the air was completely absorbed after one week. There were 12 cases (28.6%) with intracranial hematomas, but no cases had severe intracranial hemorrhage. Epidural hemoatoma was usually limited in a small area. The absorbing time of hematoma was dependent on the location of hemorrhage, with the subarachnoid hemorrhage usually absorbed completely within one week while epidural hematoma usually over three weeks. In one case with brain contusion and laceration concomitant with intracerebral hemorrhage, the hemorrhage seemed to increase within one week of injury. Five patients (10%) had subdural effusion including one male patient of 68 years of age who had the effusion extended from the left frontal area to both frontal area and both temporal area, and the other four patients were all less than 50 years of age with less and limited epidural effusion. Significant differences (P < 0.05) existed in the changes of signs with time except for those of epidural hematoma, subdural hematoma and epidural effusion (Tables 1 and 2 and Fig. 1).

**Table 1:** Comparison of frontal fracture signs with time.

Stages	Sharp fracture line	Blunt fracture line	Frontal sinus effusion	Ethmoidal sinus effusion	Sphenoid sinus effusion	Maxillary sinus effusion	Soft tissue swelling
<3d (26)	26	0	17	18	10	17	26
4–7d(28)	28	0	18	17	11	19	28
8–14d (33)	33	0	19	15	12	20	33
15–21d(27)	11	6	13	7	1	10	8
22d–2m(26)	12	12	8	2	3	2	4
>2m(8)	3	6	2	1	0	2	0
*P*	<0.001	<0.001	0.048	<0.001	0.002	<0.001	<0.001

Note: <3d, super early stage; 4–7d, early stage; 8–14d, early-to-medium stage; 15–21d, medium stage; 22d–2m, medium-to-late stage; >2 m, late stage; d, day; m, month.

**Table 2:** Comparison of frontal fracture signs with time.

Groups (n)	Pneumoceph-alus	Epidural hematoma	Subdural hematoma	SAH/contusion and laceration	Subdural edema
<3d (26)	10	5	2	6	3
4–7d(28)	3	6	3	4	2
8–14d (33)	0	6	1	1	3
15–21d(27)	0	5	0	1	2
22d–2m(26)	0	1	0	1	1
>2m(8)	0	0	0	0	0
*P*	<0.001	0.353	0.240	0.043	0.879

Note: <3d, super early stage; 4–7d, early stage; 8–14d, early-to-medium stage; 15–21d, medium stage; 22d–2m, medium-to-late stage; >2 m, late stage; d, day; m, month; SAH, subarachnoid hemorrhage.

### Orbital fractures

**Figure 2:**
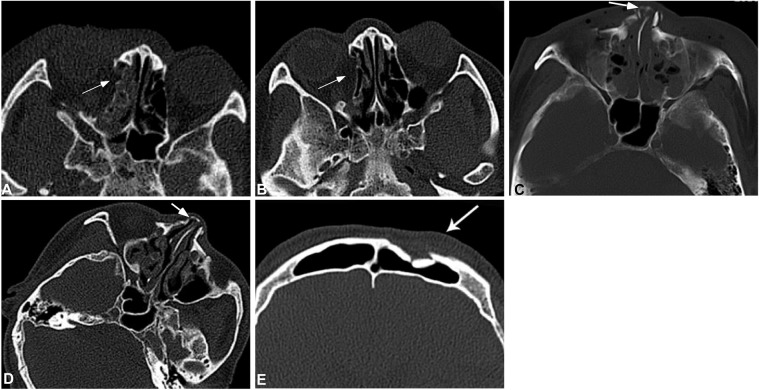
Facial fractures at different sites. A&B. A male patient aged 52 years had a traffic accident with fractures on the right orbital wall and bilateral ribs. On the day of injury, right orbital wall fracture was demonstrated with clear and sharp fracture lines, effusion in the adjacent ethmoid sinus (arrow) and soft tissue swelling (**A**). One month later (**B**), the original fracture site was smooth and depressed (arrow) with no swelling in the soft tissues. (**C**) A male patient aged 21 years was injured in the nose with nasal deformation. Computed tomographic scanning demonstrated clear a fracture line (arrow) in the nasal bone, with swelling in the adjacent soft tissues and effusion in the ethmoid sinus. (**D**) Computed tomography one month later, the fracture line became blunt with disappearance of the soft tissue swelling and effusion in the ethmoid sinus. However, the mucous membrane of the ethmoid sinus was still thickened. (**E**) A male patient aged 51 years had violent assault. Computed tomographic scanning showed old fracture lines at the left frontal sinus wall with clear fracture lines and absorption at the edge of the fracture lines (arrow).

Among all the patients, 356 had bone fractures involving the orbital wall, including 134 involving the medial orbital wall, 121 external orbital wall, 67 orbital floor, 12 superior orbital wall and 22 optic canal wall. In analysis of 126 CT scans of 40 patients with medial orbital wall fractures, soft tissue swelling existed within one week of injury in all patients, and seven patients (17.5%) had swelling of the internus. At the early stage of injury, the bone fracture line of the medial orbital wall was clear and sharp, and during two to three weeks following injury, smooth depression appeared at the original fracture site with some patients having adipose tissue and intraorbital muscle at the depression. During the study period, a significant difference (P < 0.05) existed in the signs studied except for thickness of the medial rectal muscle (Table 3 and Fig. 2A,B)

**Table 3:** Comparison of medial orbital wall fracture signs with time (n).

Groups (n)	Sharp fracture line	Smooth fracture line	Ethmoidal sinus effusion	Thickened medial rectal muscle	Orbital soft tissue swelling
<3d(30)	30	0	30	7	30
4–7d(20)	20	0	20	5	19
8–14d(26)	16	0	9	6	14
15–21d(25)	8	15	2	4	4
22d–2m(20)	0	17	1	3	2
>2m(5)	0	5	0	1	0
*P*	<0.001	<0.001	<0.001	0.947	<0.001

Note: <3d, super early stage; 4–7d, early stage; 8–14d, early-to-medium stage; 15–21d, medium stage; 22d–2m, medium-to-late stage; >2 m, late stage; d, day; m, month.

### Nasal fracture

Fifty patients had nasal fractures which were caused by high-energy impact, including traffic accidents in 23 cases, violent assault in 13 and other reasons in 14. Nineteen patients (38%) had concurrent nasal septum fractures and 12 (24%) had concurrent fractures of the frontal process of the maxilla. At the early stage, all fractures had swelling in adjacent soft tissues. The swelling gradually disappeared one week later, the fracture line became blunt three weeks later but could still be seen over two months with different degrees of bone absorption at the fracture line. A significant (P < 0.05) difference existed in the sharpness and bluntness of the fracture line (Table 4 and Fig. 2C).

**Table 4:** Comparison of nasal/maxillary frontal process fracture signs with time (n).

Groups	*n*	Sharp fracture line	Blunt/sclerotic fracture line	Soft tissue swelling
<3d	33	33	0	30
4–7d	25	25	0	21
8–14d	29	18	0	15
15–21d	27	14	9	4
22d–2m	21	3	16	0
>2m	10	0	7	0
*P*		<0.001	<0.001	<0.001

Note: <3d, super early stage; 4–7d, early stage; 8–14d, early-to-medium stage; 15–21d, medium stage; 22d–2m, medium-to-late stage; >2 m, late stage; d, day; m, month.

## Discussion

### Facial fractures and development

Facial skeletal fractures are caused by high-energy impact. During the super early and early stages, the fracture lines are usually clear and sharp with soft tissue swelling and possible paranasal sinus effusion (Tables 5–7). Two to three weeks later, the fracture line will become blunt and sclerotic due to bone absorption at the edge of the fracture. Facial fractures involving the frontal sinus or other paranasal sinuses will lead to pneumocephalus or air collection in adjacent soft tissues within three days of injury. Intracranial injury may also occur including intracranial hematoma and effusion. The subarachnoid hemorrhage will usually be absorbed within one week while the epidural hematoma over three weeks. Significant changes in the fracture lines, effusion of paranasal sinuses, soft tissue swelling and pneumocephalus are observed during the study period except for epidural and subdural hematoma, subarachnoid hemorrhage and brain contusion and laceration. Identification of the dynamic changes of facial fractures will be helpful to clinical diagnosis of fracture severity and time (Tables 5–7).

**Table 5:** Frontal bone fracture at six stages.

Signs	1st 3 stages (within 2 w)	Latter 3 stages (after 2 weeks)	P
Fracture lines (incidence)	Clear, sharp (26/26, 28/28, 33/33)	blunt and sclerotic at edges (11/27, 12/26, 3/8)	<0.05
Paranasal sinus effusion (incidence)	Effusion and mucosa thickening (62/104, 65/112, 66/132)	Decreased incidence (31/108, 15/104, 5/32)	<0.05
Soft tissue swelling (incidence)	Soft tissue swelling and mucosa thickening (26/26, 28/28, 33/33)	Decreased incidence (8/27, 4/26, 0/8)	<0.05
Gas accumulation in tissue and intracranially	Yes (10/26, 3/28, 0/33)	Disappeared over 1 week	<0.05
Subarachnoid hemorrhage (incidence)	Few and disappeared within 1 week (6/26, 4/28, 1/33)	Fewer (1/27, 1/26, 0/8)	>0.05
Epidural hematoma (incidence)	Few (5/26, 6/28, 6/33)	Absorbed over 3 weeks (5/27, 1/26, 0/8)	>0.05
Subdural hematoma	Few (2/26, 3/28, 1/33)	None	>0.05
Subdural effusion (incidence)	Few (3/26, 2/28, 3/33)	Fewer (2/27, 1/26, 0/8)	>0.05

Note: P value indicated the difference in the incidence of signs between the first and later three stages.

**Table 6:** Orbital fractures at six stages.

Signs	1st 3 stages (within 2 w)	Latter 3 stages (after 2 weeks)	P
Fracture lines (incidence)	Clear and sharp (30/30, 20/20, 16/26)	Blunt and depression with fat or muscle entrapment (15/25, 17/20, 5/5)	<0.05
Ethmoid sinus effusion	Higher (30/30, 20/20, 9/26)	Fewer (2/25, 1/20, 0/5)	<0.05
Medial rectus thickening	Higher (7/30, 6/20, 5/26)	Fewer (4/25, 3/20,1/5)	>0.05
Soft tissue swelling	Higher (30/30, 19/20,14/26)	Fewer (4/25, 2/20, 0/5)	<0.05

Note: P value indicated the difference in the incidence of signs between the first and later three stages.

**Table 7:** Nasal bone/maxillary frontal process fractures.

Signs	1st 3 stages (within 2 w)	Latter 3 stages (after 2 weeks)	P
Fracture lines (incidence)	Clear and sharp (33/33, 25/25, 18/29)	Blunt and sclerotic (9/27, 16/21, 7/10)	<0.05
Soft tissue swelling (incidence)	Higher (30/33, 21/25, 15/29)	Fewer (4/27, 0/21, 0/10)	<0.05

Note: P value indicated the difference in the incidence of signs between the first and later three stages.

### Frontal bone fracture

Frontal bones are very strong, and frontal bone fractures require greater than 800–2200 pounds of force^14^. Consequently, frontal bone fractures are usually accompanied by fractures in other bones, with possible intracranial injuries including intracranial hematoma, pneumocephalus, brain contusion and laceration and even cerebral hernia in severe cases^9,15,16^. The fracture pattern of frontal bone fracture define necessary management including open surgical, endoscopic or conservative approaches because treatment varies according to the involvement of the outflow tract of the frontal sinus^14^. CT scan without contrast is the gold standard modality of imaging and is able to show the range of fractures, involvement of the outflow tract of the frontal sinus and anterior or posterior tables of the frontal bone. However, initial assessment of fractures of the frontal bone or sinus should clarify the bony extent and involvement of other structures. Moreover, cerebrospinal fluid leakage should be assessed in detail at both the initial examination and over time. For frontal bone fractures, the acute concerns involve protection of the intracranial and orbital contents, clarification and management of concomitant injuries of the craniomaxillofacial skeleton, control of leakage of cerebrospinal fluid or rhinorrhea, prevention of wound infection after trauma, and restoration of an esthetic forehead contour^17^.

In our study, 28.6% of patients with frontal bone fractures had intracranial hematoma, which is in line with a report that one third of patients with facial fractures will have craniocerebral injury^16^. Frontal bone fractures will mostly involve the frontal, maxillary and sphenoid sinuses, resulting in sinus effusion or bleeding which will mostly be absorbed 2–3 weeks later. Air inside the sinuses can diffuse into adjacent soft tissues and even inside the skull after skull fractures, and the air can be absorbed quickly on the second day. Most air in soft tissues and pneumocephalus can be absorbed on third day. Epidural hematoma may also occur but usually in a small volume and be absorbed three weeks later. Traumatic subarachnoid hemorrhage is usually absorbed within one week, which is relatively quicker than that caused by intracranial aneurysms^18^. For patients with brain contusion and laceration, the intracranial hemorrhage and edema may be aggravated within one week of injury which should be realized. Frontal fractures may result in epidural effusion caused by cerebrospinal fluid leakage into the epidural space due to dilaceration of the arachnoid membrane. The dilacerated arachnoid membrane may form a valve under intracranial pressure resulting in continued leakage of cerebrospinal fluid into the epidural space so that the epidural effusion is difficult to be absorbed. The volume of the epidural effusion is dependent on the pressure balance across the valve. In this study, one old man had the epidural effusion increased with time probably due to decreased cerebral tissue but increased epidural space. Five other patients had relatively small amounts of epidural effusion, and no CT follow-up was performed in later period because of no increased intracranial pressure. Whether or not the epidural effusion will persist or increase with time needs further observation.

The key to differentiating new and old bone fractures lies in injury mechanism, sharpness or bluntness of fracture lines and soft tissue injury. One patient who had had old frontal bone fractures was injured once again, with bruise and hemorrhage at the injured frontal area, but he denied having been injured with frontal fractures. CT images showed blunt and sclerotic fracture lines immediately after injury with slight swelling of soft tissues (Fig. 2E). Frontal bone fractures are mostly caused by high-energy impact. In this patient who had violent assault with lower energy, the fracture line involved the frontal sinus wall but with no sinus effusion nor air collection in adjacent soft tissues, and the blunt and sclerotic fracture line did not support new fracture. So the frontal bone fractures were diagnosed as old fractures caused by traffic accidents one year ago.

### Orbital fracture

Orbital fracture is the second fracture site in the maxillofacial region secondary to nasal fracture. A good understanding of the anatomy of the orbit is very important in assessment and management of the patient with orbital fractures. Seven facial bones comprise the orbit including the frontal, lacrimal, zygomatic,maxillary, sphenoid, palatine and ethmoid bones^19^. The medial to the infraorbital canal down the floor of the orbit is very thin and most vulnerable to fracture, measuring 0.4–0.5 mm in thickness. Forces from blunt trauma can be spread to this area leading to orbital floor fracture^20^.

The most frequent fracture site is intraorbital wall fracture followed by extraorbital wall and orbital floor with rare fractures involving the superior orbital wall. The front part of the extraorbital wall is relatively thick consisting of zygomatic and frontal bones, and fractures at this site are usually caused by high-energy assault with concurrent fractures at other sites^21–23^. The ethmoid board is very thin and low-energy impact can result in fractures at this site. Intaorbital fractures are frequently accompanied by fractures in the nasal bone, the frontal process of the maxillary bone or the orbital floor^21–23^, leading to orbital wall depression. No studies to date have evaluated bony changes in the un-operated orbital bones with time even though there is extant literature which examines changes in orbital soft tissue at various time points after injury and evaluates bony changes in operated orbital fractures. What would make our study unique was its examination of changes over time in fractures which have not had surgical intervention^24^. In our study, the intraorbital wall fracture lines were clear and sharp within one week of injury, with adjacent sphenoid sinus effusion and soft tissue swelling. The intraorbital wall depression usually occurred 2–3 weeks after injury when the sphenoid effusion and soft tissue swelling had disappeared. Smooth depression at the ethmoid board usually indicates old fractures over two weeks.

### Nasal fracture

The nasal bone is the mostly affected bone in fractures of the facial structures, with the incidence of fracture as great as 40% among all facial bone fractures^25,26^. Fractures involving the nasal bone and/or the frontal process of the maxillary bone had a high incidence in our study, accounting for nearly one half of all patients (45.8%), similar to orbital fractures. This is in line with the report by other authors^23^. The nasal fractures can occur independently or with concurrent fractures in other bones^2,23,27^. The nasal bone can easily be injured because it is thin and protrudes in the face. Violent assaults can result in its fracture. Low-energy impact can lead to fractures in the frontal process of the maxillary bone, nasal septum, intraorbital and orbital floor, with the fracture often localized at the middle and lower area. High-energy impact can cause fractures in the nasal bone concurrent with fractures in other maxillofacial bones and cranial base, with mostly comminuted fracture localized in the high part of the nasal area. The fracture line of nasal bone often existed for a long time, and old fractures should be discriminated from old one. Nasal swelling, depression and deflection of the nasal bone together with injury history are the key to proper determination of nasal bone fracture and the time. Although some approaches have been suggested to manage nasal fractures for optimal functional and cosmetic outcomes, a high rate of posttraumatic deformation exists and needs open septorhinoplasty^26^. This fact requires accurate and early diagnosis of nasal fractures before correct management can be performed.

### Fracture stages

In early stages of fractures, the fracture line is usually clear and sharp, and indirect signs of fracture include swelling, effusion and air collection in adjacent soft tissues, paranasal sinus effusion and craniocerebral injury. Pneumocephalus and air collection in adjacent soft tissues will usually absorbed within three days of injury, and soft tissue swelling and sinus effusion will be improved or disappear during one and two weeks. The fracture line at early stages is clear and sharp and will become blurred and blunt with bone absorption 2–3 weeks later. For some parts of bone which are difficult to be healed, the fracture line will become blunt and sclerotic. The fracture site will become stable over 3 weeks, and no apparent changes at the fracture site will be observed over two months which can be used as a time point. So, this study divided the observing time into six stages as super early (<3d), early (4–7d), early-to-medium (8–14d), medium (15–21d), medium-to-late (22d–2 months) and late stage (>2 months).

The healing process of craniofacial bones is through membranization and is slow. Some fractures can still be observed even after a long time, and the fractured bones are connected by fibrous tissues, with bone absorption at the fracture site. The nasal bone may have a high incidence of fibrous connection at the fracture site for a long time. The sharpness of the fracture line of the nasal bone and surrounding soft tissue swelling can be used to judge the time of fracture, and immediate CT scanning will provide these signs. The peak time for soft tissue swelling is usually at 3–5 days after injury, and for patients with difficulty of diagnosis, CT scanning can be performed repeatedly based on the changes of fracture lines. Two to three weeks later, the fracture line will become blurred and blunt with disappearance of soft tissue swelling.

### CT scanning

Craniofacial bones have complex anatomy and are usually overlapped with one another, which is difficult for evaluation with plain radiography. CT scanning is frequently applied for craniofacial fractures while magnetic resonance imaging is not sensitive to bone fractures except for clinical suspect of axonal injury. During CT scanning, proper scanning parameters and reconstruction approaches are very important for displaying bone fractures. For fractures at thin bones, thicker slices or intervals may miss the fracture due to partial volume effect or miss the sharp edge of the fracture line, and consequently, thin slices ≤1 mm for reconstruction will be needed for observing fractures at the sphenoid and nasal bones. For nasal bone fractures, coronary scanning and reconstruction are also very important for proper diagnosis. In selection of reconstruction algorithm, the sharping effect in high resolution bone algorithm can display more clearly the fracture edge, whereas common algorithm reconstruction may decrease the sharpness of fracture lines and consequently its role in evaluating new and old fractures. Multiplanar reconstruction plus three-dimensional reconstruction can directly display the whole fracture line and are very useful for differentiating nasal fractures, sutura and holes.

## Limitations

This study may have some limitations. One is of retrospective nature, which may affect its effect because of possible bias and limited data. Another is that the sampled patients were all of the Chinese ethnicity involving no other ethnicities, which limit its generalization to other races because the facial structures or anatomy of one race may be different from another. A third limitation is that it was a one center study with no other medical centers involved since one center study may cause some statistical bias. The CT scanning parameters were different in some patients or at different time points, which may thus affect the imaging presentations. Some patients did not have radiological check-up at all the six stages, and multiple fractures existed in one patient, which may all affect the imaging results. Few patients had radiological examinations during latter stages. In the future, multiple centers will have to be involved to a prospective study with more ethnicities and patients participating in the study so as to eliminate some limitations inherent to one-center retrospective studies and to generalize the outcome to more ethnicities.

## Conclusions

In conclusion, facial fractures have some dynamic alterations with time and identification of these characteristics may help reaching a correct clinical diagnosis with regard to fracture severity and time. Radiologists/doctors can use this information about radiographic changes in facial fractures with time to help determine whether the fractures are acute or old and help guide treatment/surgical planning in conjunction with the clinical exam and history.
